# Natural Language Processing Chatbot–Based Interventions for Improvement of Diet, Physical Activity, and Tobacco Smoking Behaviors: Systematic Review

**DOI:** 10.2196/66403

**Published:** 2025-06-11

**Authors:** Jing Chen, Run-Ze Hu, Yu-Xuan Zhuang, Jia-Qi Zhang, Rui Shan, Yang Yang, Zheng Liu

**Affiliations:** 1 Department of Maternal and Child Health, School of Public Health, Peking University, No. 38, Xueyuan Road, Haidian District, Beijing, 100191, China; 2 Department of Preventive Medicine, School of Public Health, Peking University, Beijing, China

**Keywords:** NLP-chatbot, intervention, diet, physical activity, tobacco smoking, RCTs, natural language processing, randomized controlled trial

## Abstract

**Background:**

The rapid development of artificial intelligence technology has enabled chatbots to increasingly promote health-related behaviors, addressing the high demand for human resources in traditional interventions. Several systematic reviews have been conducted in this area. However, the existing reviews have not focused on the rigorously designed randomized trials of the state-of-the-art chatbots (interacting with users through unconstrained natural language), thus calling for an updated review.

**Objective:**

We aimed to explore the effects of natural language processing (NLP) chatbot–based interventions on improving diet, physical activity, and tobacco smoking behaviors in the general population and to evaluate the chatbot use behaviors during the implementation process.

**Methods:**

We comprehensively searched 12 databases or registers for eligible studies published from January 1, 2010, until July 16, 2024, and obtained a total of 6301 studies. We included randomized controlled trials (RCTs) that used NLP-chatbots to promote diet, physical activity, or tobacco smoking behaviors among adults or children. Due to considerable heterogeneity across the included studies, we adopted the synthesis without meta-analysis guidelines and summarized the effectiveness of NLP chatbot–based interventions. We used the new evidence-mapping method (bubble plot) to visualize the results. We also described the results related to the changes in diet, physical activity, or tobacco smoking behaviors (eg, change of BMI and stage of change). To evaluate the implementation process of the intervention, we summarized users’ interaction with NLP-chatbots and their feelings (eg, satisfaction) about NLP-chatbot use. Additionally, we assessed the risk of bias of studies using the RoB 2.0 (Risk of Bias; The Cochrane Collaboration) tools.

**Results:**

We finally included 7 RCTs. Concerning dietary and physical activity behaviors, the effectiveness of NLP chatbot–based interventions was inconsistent among adults, while no evidence of effect was observed among children. Concerning tobacco smoking behaviors, the included studies showed consistent evidence of improving this behavior among adults. Regarding the risk of bias of the changes in diet, physical activity, and tobacco smoking behaviors, 2 of 3, 2 of 4, and 1 of 2 studies had a high risk of bias, respectively, while the remaining had a low risk of bias. Concerning the interactions with NLP-chatbots, studies showed an overall high percentage of general interaction between users and NLP-chatbots, but not a satisfactorily high percentage of interactions specific to health behaviors. Concerning feelings about NLP-chatbot use, users showed a positive impression of NLP-chatbot use, feeling it was useful, credible, and financially feasible.

**Conclusions:**

NLP chatbot–based interventions were beneficial for adults’ tobacco smoking behaviors, but no such evidence was found on diet or physical activity behaviors among adults or children. More RCTs with larger samples and lower risk of bias are urgently needed to enhance our findings in the future.

## Introduction

Worldwide, physical inactivity, unhealthy diet, and tobacco use are the 3 major behavioral risk factors responsible for chronic, noncommunicable diseases [1]. The prevalence of these 3 factors was high in the population. Concerning physical inactivity, a significant proportion of populations fail to meet recommended activity levels [2]. Specifically, 81% of school-aged children (11‐17 y) did not achieve the minimum requirement of 60 minutes of moderate-to-vigorous physical activity daily, while 27.5% of adults fell short of the weekly recommendation of either 150 minutes of moderate-intensity activity, 75 minutes of vigorous-intensity activity, or an equivalent combination of both [2,3]. Concerning unhealthy diets, the vegetable supply was insufficient to meet the recommendations in 61% of the countries [4]. Specifically, most African and South American populations, as well as a part of Asian and North American populations, did not have sufficient (200‐250 g per day) vegetable intake [5]. Additionally, most African and parts of Asian populations did not have sufficient (200 g per day) fruit intake [6]. Concerning tobacco smoking, the global smoking prevalence rate among people aged 15 years and older was 16.7% in 2022, despite significant past achievements in tobacco smoking control [7]. These high prevalences also posed challenges to global actions for better human health. Physical inactivity and unhealthy diet have paralleled the rising prevalence of overweight or obesity (BMI≥25 kg/m²) from 38% in 2020 to 46% in 2030 according to the published trends from 1975 to 2016 [8], making it challenging to reverse the obesity epidemic and achieve the World Health Organization’s target of “no increase on obesity levels“ by 2025 (based on 2010 levels). Tobacco smoking remains the most prevalent form of tobacco use. The persistently high tobacco smoking prevalence poses a significant challenge to achieving the World Health Organization’s target of a 30% reduction in tobacco use from 2010 to 2025 [9].

To achieve a higher level of human health, actions are urgently needed to reverse these unhealthy behavioral risk factors. However, most conventional behavioral interventions cannot be faithfully implemented at the population scale due to the high demand for human resources. Thanks to the rapid development of artificial intelligence (AI) technology, chatbots have become a viable alternative to delivering resource-intensive, conventional behavioral interventions. Chatbots, also called intelligent dialogue systems or conversational agents, are machine agents that are designed to converse with humans using natural language through text or voice interactions, which can be classified into constrained and unconstrained ones. The former refers to those that only interact with the user through selection questions with fixed predefined options, while the latter can engage in free human-like dialogue with users and interact with users through unconstrained natural language. Notably, the recent rapid development of natural language processing (NLP) has led to obvious advancements in the capabilities from constrained ones to unconstrained ones. Therefore, it is urgent to summarize the development potential of chatbots based on NLP in improving individuals’ health behavior.

There is evidence that chatbots have great potential to persuade, support, and promote individuals to change health-related behaviors and can be used to improve diet, physical activity, and tobacco smoking behaviors [10-12]. Previous systematic reviews involving qualitative summary or quantitative description through meta-analysis showed that chatbot-based interventions can improve physical activity, increase fruit and vegetable consumption, and enhance the individuals’ intention to quit smoking [13-15]. However, few previous reviews have strictly distinguished between constrained and unconstrained chatbots [13,16-20]. Besides, concerning study design, reviews have included both randomized and nonrandomized studies, resulting in a variable strength of evidence [14,21,22]. The existing reviews were also limited in the search strategies and risk of bias assessment [23].

Bearing these research gaps in mind, we aimed to systematically review the existing randomized trials of the present topic and rigorously evaluate the evidence quality to (1) explore the effects of NLP chatbot–based interventions on improving diet, physical activity, and tobacco smoking behaviors in the general population; and (2) evaluate the NLP-chatbot use behaviors during the implementation process. Findings from this study would pave the way for the improvement of the NLP chatbot–based intervention for health behavior change.

## Methods

### Study Design

We conducted the systematic review following the guidelines of the PRISMA (Preferred Reporting Items for Systematic Reviews and Meta-Analyses) [24]. The protocol was registered in the PROSPERO (International Prospective Register of Systematic Reviews) on December 21, 2023 (CRD42023492013).

### Search Strategy

We conducted an extensive electronic search to identify all randomized controlled trials (RCTs) that reported outcomes measured by changes in behaviors in diet, physical activity, or tobacco smoking after the NLP chatbot–based intervention.

Eleven databases (PubMed, Embase, ACM Digital Library, Web of Science Core Collection, PsycINFO, IEEE, CINAHL Plus with Full Text, Cochrane Library, Scopus, Information Science & Technology Abstracts, and China National Knowledge Infrastructure) and one register (ClinicalTrials.gov) were systematically searched until July 16, 2024. The search strategy used a combination of keywords and Medical Subject Headings terms from the following 5 aspects: chatbot (eg, chatbot, AI agent, or conversational agent), diet (eg, diet or nutrition), physical activity (eg, physical activity, exercise, or sedentary behavior), tobacco smoking (eg, smoking cessation or smoking habit), and lifestyle (eg, weight control or lifestyle). The Boolean operator “OR” was used in each aspect, and between the last 4 aspects, and “AND” was used to combine “chatbot” with the other 4 aspects. The detailed search strategy for each database is reported in Multimedia Appendix 1. There were no restrictions on publication status or language. Due to the rapid development of conversational agents in recent decades, we limited the included studies to those published in or after 2010.

### Study Selection

Table 1 presents a summary of the inclusion and exclusion criteria of the study characteristics based on the PICOS framework (eg, populations or participants, interventions and comparators, outcomes, and study designs or types). Two reviewers (JC and YXZ) independently conducted a 2-stage study selection process: initially screening titles and abstracts, followed by a thorough examination of the full texts in the second stage. Discrepancies were resolved through consultation with a third review author (ZL).

**Table 1.** Summary of inclusion and exclusion criteria.

PICOS^a^	Inclusion criteria	Exclusion criteria
Population	General population	None
Interventions	NLP^b^ chatbot–based interventions that enable natural language conversations with users for improving diet, physical activity, or tobacco smoking behaviors	Chatbots that cannot understand or generate natural language conversations
Comparators	Active controls^c^ or no-intervention controls^d^	None
Outcomes	Primary outcomes^e^: changes in behaviors in diet, physical activity, or tobacco smoking Secondary outcomes: the following 2 aspects are included: Effectiveness-related outcomes: change of obesity-related outcomes (eg, BMI and weight), stage of change of diet, physical activity or tobacco smoking behavior (eg, precontemplation, contemplation, action, or maintenance), or change of mediators^f^ (eg, motivation) Implementation of NLP chatbot–based interventions: chatbot use behaviors (eg, duration, frequency, or interaction), facilitators and barriers to chatbot use (eg, satisfaction, appreciation, or feelings or acceptability), and safety issues^g^ (eg, adverse events)	Studies that report only chatbot infrastructure or algorithm designs, or that did not report any of the primary outcomes
Study designs	Individual-randomized or cluster-randomized parallel-group controlled trials	Observational studies, nonrandomized controlled studies

a PICOS: Population, Interventions, Comparators, Outcomes, and Study Designs.

b NLP: natural language processing.

c Active controls refer to providing participants with intervention measures other than the chatbot.

d No-intervention group includes those groups that have not implemented any special intervention measures or the wait-list control groups that have not yet received intervention in this study.

e Primary outcomes are measured by various means. For example, daily physical activity was recorded through a portable accelerometer, the intake of various types of food was investigated through a questionnaire, and the concentration of carbon monoxide in exhaled gas was measured to detect smoking behavior.

f Potentially mediate the effect of natural language processing–chatbot-based interventions for the improvement of diet, physical activity, and tobacco smoking behavior.

g Safety issues refer to unintended adverse events and the privacy protection of participants.

### Data Extraction

Information extracted from the studies included participant characteristics (mean age, sex, country, race, income, education, baseline BMI score, frequency of smoking, stage of behavior change, recruitment setting, or sample size), study design, NLP-chatbot (referred to as the “chatbot” in the following sections) characteristics (theoretical framework, media or technology, dialogue initiative, input modality, output modality, or task-oriented), intervention measures (chatbot only or not, duration, or length and frequency), and results (primary outcomes and second outcomes) along with barriers and facilitators to the use of chatbot. Two researchers (YXZ and RZH) independently extracted data from the included studies and consulted with the other 2 researchers (JC and ZL) when discrepancies arose.

### Data Synthesis

The primary outcome was the difference in changes in physical activity, diet, and tobacco smoking from baseline to follow-up between the intervention (NLP-chatbot) and control groups. Due to heterogeneity in outcome measurement and varied indicators across studies (eg, daily active time, sedentary behavior time, or average number of daily steps), we refrained from conducting a meta-analysis. Following the Cochrane Handbook (Chapter 12: synthesizing and presenting findings using other methods) [25], we qualitatively summarized the effectiveness of NLP chatbot–based interventions instead. We evaluated the intervention effectiveness estimates for each study individually. Categories of intervention effectiveness were differentiated into 4 groups based on the proportion of effective indicators in the primary outcome: (1) if all indicators were effective, it was considered effective (E); (2) if the effective ratio exceeded 50%, it was considered to be probably effective (PE); (3) if the effective ratio was less than 50%, it was considered not to be probably effective (PNE); (4) if the effective ratio was 0, it was considered not effective (NE). An indicator was considered effective when there was a statistically significant difference (*P* value <.05) in prepost changes between the intervention and control groups.

The following are the specific methods for judging whether the primary outcome indicators were effective. For each primary outcome, when studies reported *P* values for between-group comparisons (intervention vs control), these *P* values were prioritized in our analysis. For studies that did not report between-group *P* values directly, we calculated them using independent samples *t*-tests. We used the original data provided in the papers, including sample sizes, prepost mean differences, and SE for both intervention and control groups. When change score data were unavailable, between-group differences in postintervention values were analyzed instead, with the assumption of baseline equivalence justified by randomized study design. For studies reporting both baseline data and results at multiple follow-up time points, measurements across all follow-up time points were integrated using an equally weighted average method. The composite result was then compared against the baseline data for analysis. For the 3-arm intervention studies included, the intervention groups were both NLP chatbot–based interventions with slight differences in form. Therefore, we integrated the 2 intervention groups into 1 group and compared it with the control group. For the studies based on families, we considered adults and children separately. We examined whether each primary outcome indicator was effective and then summarized the possible effectiveness of the intervention for adults and children, respectively.

To present the results intuitively, we adopted a new evidence-mapping method to summarize the findings [26,27]. Specifically, we used bubble plots to visualize the whole patterns of results, considering the following factors in an intergrade manner: the outcomes (diet, physical activity, or smoking behaviors), sample size, population (adults and children, adults only, or children only), effectiveness of intervention, and risk of bias of studies.

For our secondary outcomes, we provided a descriptive summary of the effectiveness-related outcomes and the implementation of the NLP chatbot–based intervention. We used appropriate metrics, such as proportion, to quantify the frequency and duration of chatbot use and the number of interaction turns per dialogue in the included studies. We also focused on the acceptability of chatbots and therefore summarized the feelings of participants in the corresponding studies using them.

### Assessment of the Outcome Quality and Evidence Certainty

Risk of bias assessment was performed exclusively for primary outcomes. For individual RCTs, we used the RoB 2.0 (Risk of Bias) tool [28], while for cluster RCTs, we used the RoB 2.0 tool for cluster-randomized trials [29]. The assessment for outcomes from individual RCTs contains the following five domains: (1) bias in the randomization process; (2) bias in deviation from intended interventions; (3) bias in missing outcome data; (4) bias in outcome measurement; and (5) bias in the selection of the reported result. Each domain was rated as having a high, low, or some concerns about the risk of bias. The assessment for outcomes from cluster RCTs was mostly consistent with that for individual RCTs, except for the first domain specified into (1) bias in the randomization process and (2) bias arising from the timing of identification or recruitment of participants.

Based on the biases identified in the aforementioned 5 aspects, we further assessed the overall bias risk of each primary outcome and rated them as: low risk of bias, some concerns, or high risk of bias. We then generated a bias risk assessment plot to present these results. Two researchers (YXZ and RZH) independently conducted the assessment of the included studies’ bias, while a third author (JC) facilitated discussions to achieve consensus on discrepancies.

### Ethical Considerations

Human subject ethics review approvals or exemptions: our study was reviewed and approved by the Peking University Institutional Review Board (IRB00001052-22091).

Informed consent: the original studies included in this review have obtained informed consent. Therefore, informed consent does not need to be obtained again for this review (secondary analysis).

Privacy and confidentiality: the data used in this study had been anonymized.

Compensation details: not applicable.

## Results

### Literature Search

**Figure 1.**
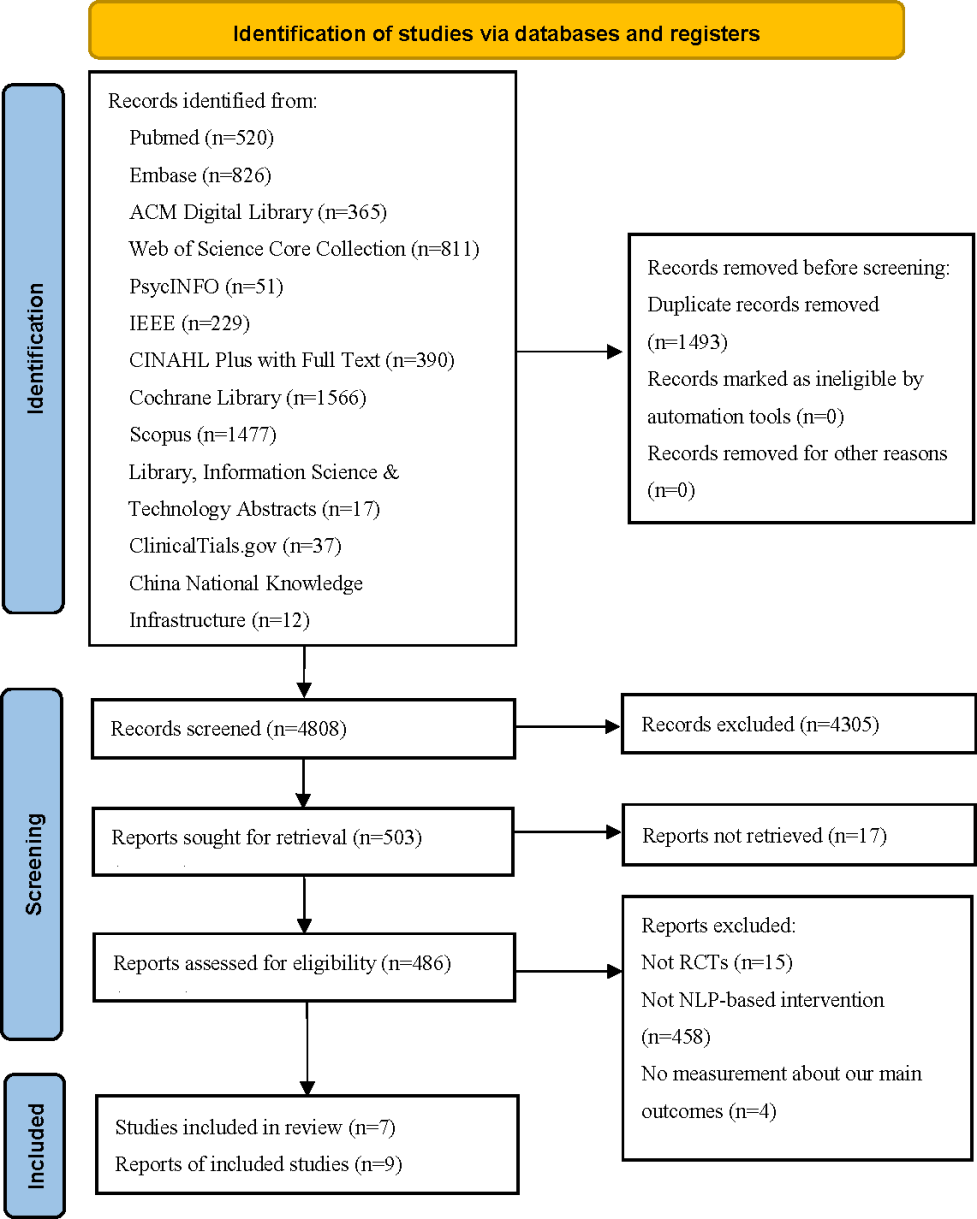
Flowchart of the study selection process. NLP: natural language processing; RCT: randomized controlled trial.

Figure 1 shows the flow of study selection. Our search yielded 4808 records after excluding duplicates. After screening the titles and abstracts, we assessed the full-text articles of the remaining 486 records. A total of 7 studies were finally included.

### Characteristics of Included Studies

Table 2 shows the primary characteristics of the 7 included studies, which were conducted in America (n=2) [30,31], Northern Ireland (n=1) [32], Spain (n=1) [33], Dutch (n=1) [34], China (n=1) [35], and Saudi Arabia (n=1) [36] from 2013 to 2022. Two studies focused only on physical activity [31,34], 1 study focused only on diet [36], 2 studies focused only on smoking [33,35], and 2 studies focused on both physical activity and diet [30,32]. In 5 studies, the participants were only adults [31,33-36], while the other 2 studies, which were family-based, contained both adults and children [30,32].

**Table 2.** Characteristics of participants among the 7 included studies.

ID	Study	Mean age (SD) years or range	Sex (female), (%)	Country, % (n/N)	BMI	Tobacco use	Baseline sample size	Attrition and rate	Follow-up sample size
1	Wright et al (2013) [30]	Children 10.3 (1.1), parents 40 (9.1)	Children 42, parents 96	USA (100)	Children 25.7 (2.1), parents 34 (6.7)	—^a^	Families n=50, children n=50, parents n=50	Attrition n=7, rate=14%	Families n=43, children n=43, parents n=43
2	Hassoon et al (2021) [31]	62.1 (9.8)	90	USA (100)	32.9 (5.0)	—	Adults n=42	Attrition n=0, rate =0%	Adults n=42
3	Carlin et al (2021) [32]	Phase 1: adults 40.5 (5.4), children 9.1 (2.0); phase 2: adults 38.9 (5.2), children 7.9 (2.0)	Phase 1: adults 10 (91), children 9 (56); phase 2: adults 11 (73), children 8 (44)	Western Trust area of Northern Ireland (100)	Phase 1: children —, adults 35.0 (6.4); phase 2: children—, adults 29.1 (4.9)	—	Phase 1: families n=11, parents n=11, children n=16; phase 2: families n=15, parents n=15, children n=18	Phase 1: attrition n=3, rate =27.3%; phase 2: attrition n=0, rate =0	Phase 1: families n=8, parents and children —; phase 2: families n=15, parents n=15, children n=18
4	Olano-Espinosa et al (2022) [33]	49.8 (10.82)	59.30	93.8 (481/513) were Spanish	—	10.1% (52/513) of patients reported moderate or high dependence on nicotine with Heavy Smoking Index values of 4‐6 points and average consumption of 16.5 cigarettes/day (SD 7.75).	Adults n=513	Attrition n=281, rate =54.8%	Adults n=232
5	Friederichs et al (2014) [34]	Baseline 42.9 (14.5), follow up 45.3 (14.2)	Female: baseline 60.4 (578/958), follow-up 57.8 (289/500)	Dutch (100)	—	—	Adults n=958; attrition n=458, rate =47.8%	n=958; attrition n=458, rate =47.8%	Adults n=500
6	Wang et al (2018) [35]	Intervention group 32.8, control group 33.1	40.4	China (100)	—	—	Adults n=401	Attrition n=114, rate =28.4%	Adults n=287
7	Alghamdi and Alnanih (2021) [36]	—	—	Saudi Arabia (100)	—	—	n=60	Attrition n=0, rate =0%	n=60

a Not applicable.

As shown in Tables3  4, among the included studies, 5 were 2-arm individual RCTs [30,32,33,35,36], while 2 used a 3-arm design [31,34]. The control groups of the 5 two-arm studies varied, including 4 no-intervention groups and 1 active control group. In the active control group, the participants received information passively, and the process did not involve chatbots or interactions with other participants. In both 3-arm RCTs, 2 groups received distinct NLP-chatbot interventions, while the third served as a control. Specifically speaking, in 1 study [34], 1 intervention group used a motivational interviewing chatbot with an avatar for web-based physical activity guidance, while the other used a simpler avatar-based chatbot; the control group received no intervention. Another study [31] compared 2 AI coaching methods: voice-assisted delivery via a smart speaker (MyCoach) and text-based delivery (SmartText), with the active control group receiving standard cancer education materials.

**Table 3.** Characteristics of the study designs of the 7 included studies.

ID	Study	Arm	Method of randomized parallel controlled trials	Classification of chatbot				
				Media or platform of technology	Dialogue initiative (user, system, or mixed)	Input modality	Output modality	Task-oriented (yes or no)
1	Wright et al (2013) [30]	2	Cluster-randomized	Telephone	Mixed	Spoken	Spoken	Yes
2	Hassoon et al (2021) [31]	3	Individual-randomized	Smart speaker	Mixed	Spoken	Spoken	Yes
3	Carlin et al (2021) [32]	2	Cluster-randomized	A smart speaker (Echo Dot)	Mixed	Spoken	Spoken	Yes
4	Olano-Espinosa et al (2022) [33]	2	Individual-randomized	Telegram, a widely used messaging app	Mixed (bidirectional)	Written	Written	Yes
5	Friederichs et al (2014) [34]	3	Individual-randomized	Website	N/A^a^	Written	Written	Yes
6	Wang et al (2018) [35]	2	Individual-randomized	Software (combined with WeChat)	Mixed	Written	Written	Yes
7	Alghamdi and Alnanih (2021) [36]	2	Individual-randomized	App (WhatsApp, social network)	N/A	Written	Written	Yes

a N/A: not applicable.

**Table 4.** Characteristics of the interventions of the 7 included studies.

ID	Study	Intervention type	Length， frequency	Measures (brief)			Chatbot only in intervention measures
Intervention group		Control group
1	Wright et al (2013) [30]	PA^a^ and diet	12 wk, twice a week	HEAT^b^ (telephone calls twice a week delivered by an automated IVR^c^ system)	—^d^	No intervention (wait-list control)	Yes
2	Hassoon et al (2021) [31]	PA	4 wk, —^d^	Voice-assisted AI^e^ coaching delivered by smart speaker (MyCoach)	Autonomous AI coaching delivered by text (SmartText)	Received written information	Yes
3	Carlin et al (2021) [32]	PA and diet	12 wk, —	Receive an intelligent personal assistant	—	Continue as usual	No (phase 1: SWEET^f^ program)
4	Olano-Espinosa et al (2022) [33]	Smoking behavior	6 mo, —	Chatbot: Dejal@bot	—	Usual clinical practice	Yes
5	Friederichs et al (2014) [34]	PA	—, 1-time	A web-based PA intervention based on MI^g^ with an avatar (AVATAR)	A content-identical intervention without an avatar (TEXT)	No intervention	Yes
6	Wang et al (2018) [35]	Smoking behavior	2 mo, —	Conversational agents (in a WeChat group talk with each other and conversational agent server by announcements, sharing, reminders, and responses)	—	Active control (in a WeChat group, but only received smoking cessation information and tips without social support or interactions with other participants)	No
7	Alghamdi and Alnanih (2021) [36]	Diet	90 d, —	Proposed chatbot	—	No intervention	Yes

a PA: physical activity.

b HEAT: healthy eating and activity today.

c IVR: interactive voice response.

d Not applicable.

e AI: artificial intelligence.

f SWEET: safe wellbeing eating and exercise together.

g MI: motivational interviewing.

More than half of the studies (n=4) used chatbots with written input modality [33-36], while the remaining 3 studies used those with spoken input modality [30-32]. All 7 studies used chatbots only using simplex output modality, spoken or written. Three studies mentioned the adoption of theoretical frameworks including “Motivation Interview,” “Social Cognitive Theory,” and “The Chronic-Disease Extended Model” [30,34,36]. The duration of interventions ranged from 1 time to 6 months.

### Effectiveness of NLP Chatbot–Based Interventions on the Changes of Behaviors in Diet, Physical Activity, or Tobacco Smoking

Concerning diet behaviors, a study (n=54) conducted only among adults indicated that NLP chatbot–based intervention was effective [36]. Two studies (n=43 and n=22, respectively) conducted in family units suggested that NLP chatbot–based intervention did not effect children’s diet behaviors, while the results were probably not effective and not effective respectively for adults’ diet behaviors [30,32].

Concerning physical activity, both studies (n=500 and n=42, respectively) showed positive results from the intervention [31,34]. Specifically, NLP chatbot–based intervention groups showed an increased average number of daily steps or number of weekly days with at least 30 minutes of moderate physical activity and daily steps than the control groups. However, 2 other small-sample studies (n=43 and n=22, respectively) conducted on both adults and children did not show evidence of intervention effectiveness [30,32].

Concerning smoking behavior, 2 studies (n=232 and n=287, respectively) showed that NLP chatbot–based intervention can improve smoking behavior in adults [33,35].

Please see the specific assessment of results in 3 primary outcomes, as well as secondary outcomes of 7 studies in Table S1 in Multimedia Appendix 2.

**Figure 2.**
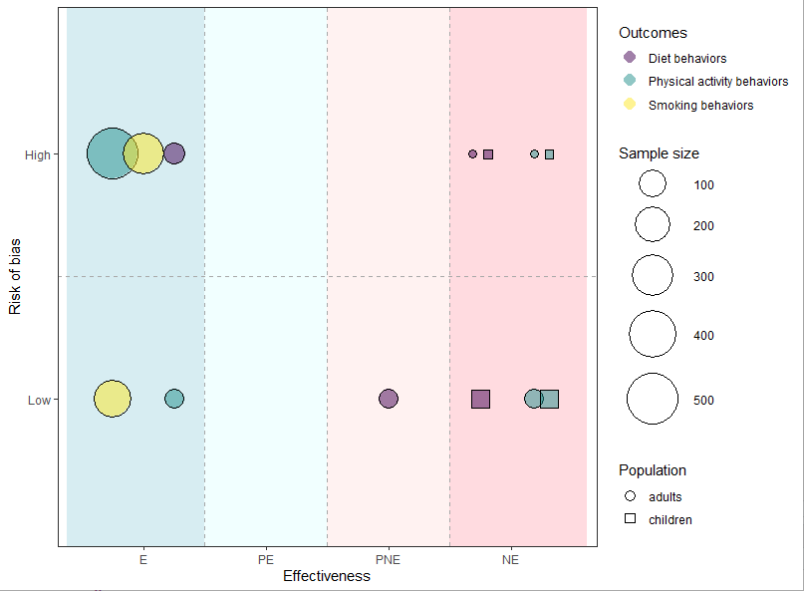
Bubble chart of the effectiveness of NLP chatbot–based interventions. E: effective; NE: not effective; NLP: natural language processing; PE: possibly effective; PNE: possibly not effective.

Figure 2 shows the bubble plot displaying the effectiveness of NLP chatbot–based interventions on the changes in diet, physical activity, and smoking behaviors, with the bubble color, size, and shape representing the outcomes, sample size, and population, respectively. To aid in understanding, we have included an example of a bubble chart for reference. The pale green bubble in the upper left corner of Figure 2 corresponds to a study involving 500 adults. This study falls into the final effectiveness category of E (effective) due to its finding that NLP-chatbots had impacts on the changes in all of the physical activity behaviors. However, it is important to note that the outcome of this study indicated a high risk of bias.

### Effectiveness of NLP Chatbot–Based Interventions on the Effectiveness-Related (Secondary) Outcomes

One study [33] compared the quality of life between the NLP chatbot–based intervention group and the control group at baseline and follow-up, and no differences were observed at either time.

Another [30] study compared changes in BMI (for adults and children), BMI percentile (for children), and BMI *z* scores (for children) between the NLP chatbot–based intervention group and the control group from baseline to follow-up, but no significant differences were observed.

Additionally, a study [36] assessed participants’ perceived difficulty in adhering to the dietary treatment plan. At baseline, 16.67% of the intervention group and 20% of the control group reported challenges in committing to the dietary treatment plan. After the NLP chatbot–based intervention, a significantly higher proportion of the intervention group (46.67%) reported no adherence difficulties, whereas only 6.67% of the control group reported no committing difficulties. The study further proposed a four-stage model of patient adaptation to chronic diseases: (1) be conscious of the need, (2) be ready to deal with the disease, (3) feel confident in dealing with the disease, and (4) stick to the plan. According to this change phase, this study investigated the proportion of different change stages among the participants in the NPL-chatbot intervention group and the control group. It was found that in the intervention group, the proportion of participants in phase 1 before the intervention was 30%, and there was no one in phase 1 after the intervention, while the proportion of those in phase 4 was 43.33%. In the control group, the proportion of participants in phase 1 before the intervention was 30%, and there were 26.67% of participants in phase 1 after the intervention, while no one was in phase 4.

### Implementation of NLP Chatbot–Based Interventions

For this review, 4 included studies assessed the implementation of NLP chatbot–based interventions. A study conducted a 2-phase family-based intervention including both adults and children [32], aiming to (1) assess the acceptability of chatbot technology for promoting and maintaining physical activity and other health-related behaviors in families attending a community-based obesity prevention project, and (2) further assess the acceptability of chatbot intervention in potentially eligible families (not restricted to those attending the project) at the second phase. The intervention time for the 2 stages was the same, lasting 12 weeks. The study found lower interaction times in phase 1 than in phase 2 (65 vs 312 times) but a higher relevant interaction rate in phase 1 (42% vs 11%). The relevant interaction rate was the rate of interactions related to diet, physical activity, or well-being. This indicated that it is possible to actively interact with chatbots in populations without a strong need for health interventions, but it also suggested that encouraging households to purposefully use the device for health-related interactions is a challenge. Another study conducted in America with a sample size of 50 dyads of parent and child aiming to improve family diet behaviors, found that a high percentage of children (81%) and parents (76%) interacted with chatbots at least once [30]. The mean number of calls for parents and children was 9.1 (SD 5.2) and 9.0 (SD 5.7).

A study aiming to improve smoking secession found that the interaction time of the chatbot intervention group was much higher than that of the control group of usual clinical practice (121 minutes vs 21.2 minutes, *P*<.001), and the number of interactions was also much higher, too (45.56 vs 2.92, *P*<.001) [33]. Within the intervention group, those who successfully quit smoking interacted with chatbots much more frequently than those who did not successfully quit smoking. Another study focusing on helping people quit smoking through group chats found that, compared to only receiving smoking cessation information and tips in group chats without social support or interactions with other participants and chatbots, significant increments of active conversations (455/341, increased by 33%) and the number of messages (248/1328, increased by 87%) were found when the chatbot was involved in the group chats [35].

Two included studies measured participants’ feelings about chatbot use, and both showed positive results. A web-based research conducted in Dutch with a large sample size of 958 participants measured participants’ appreciation for the intervention from entertainment, trustworthiness, and overall appreciation score domains, and found that participants had a very positive impression of chatbot use in all 3 domains [34]. A study conducted through phone calls between participants and chatbots had similar findings that more than 75% of those who had made calls with the chatbot to gain assistance with diet and physical activity agreed that chatbots were useful, credible, financially feasible, and really helped them eat healthy foods [30].

Two included studies reported the results regarding safety issues. A study stated that for the privacy protection of the research subjects, the search history information of the NPL-chatbot used in the research was strictly kept confidential, and during the intervention period, this device was not used for any other purposes [32]. A study emphasized that no adverse events occurred during the intervention period [31].

### Assessment of Risk of Bias

**Figure 3.**
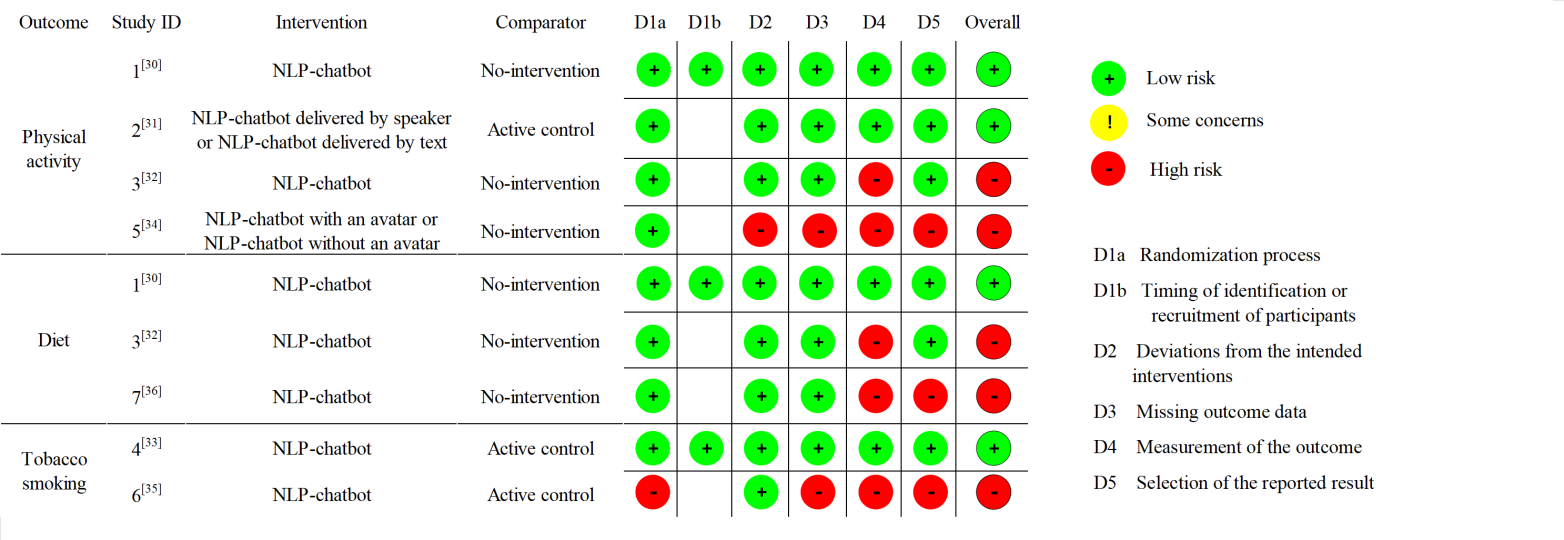
Risk of bias (RoB2 and ROB2_cluster tool) quality assessment [30,36]. NLP: natural language processing; RoB: Risk of Bias.

Figure 3 shows the result of the assessment of the risk of bias. For the changes in diet behaviors, 2 trials had a high risk of bias [32,36], and 1 trial had a low risk of bias [30]. For the changes in physical activity, 2 trials [32,34] had a high risk of bias, while the remaining 2 trials [30,31] had a low risk of bias. For the changes in tobacco smoking behavior, 1 trial [35] was judged to be at a high risk of bias, while 1 trial had a low risk of bias [33].

## Discussion

### Principal Findings

Our study was the first systematic review specifically dedicated to RCTs using NLP-chatbots for health behavior interventions related to physical activity, diet, and tobacco smoking. This focus strengthened the evidence for the results in a relatively strong manner and with the novelty prominent.

The results of the studies on dietary behavior included in our review were inconsistent. Consequently, it was ultimately impossible to clearly determine the impact of chatbots on dietary behavior. It differed slightly from those in previous reviews. One meta-analysis [14] found that chatbot intervention had a significant impact on increasing the intake of fruits and vegetables. Another review [19] showed that participants in the intervention group showed a higher self-reported willingness to reduce consumption of red and processed meat within 2 weeks compared to the control group. These 2 reviews included both RCTs and quasi-experimental studies and did not limit the types of constrained or unconstrained chatbots, differing from the eligibility criteria used in our review. Different inclusion criteria might lead to inconsistent results about the effectiveness of chatbot intervention on the changes in diet behaviors.

Among the 4 studies incorporated into this review, 2 studies discovered that chatbots exerted a positive influence on physical activity. In contrast, the other 2 studies did not observe such an effect, presenting complex and inconclusive results. Some reviews [14,19,22] have reported the positive impacts of chatbots on physical activity behaviors. However, a review [37] specifically focusing on teenage participants aged 10 to 19 years revealed limited evidence regarding the feasibility of chatbots in promoting such behaviors. This review [37] also indicated that in only 40% (2/5) of the studies, the subjects were satisfied with the application of chatbots in interventions, suggesting that there is insufficient evidence for the acceptability of chatbots. The low satisfaction level might lead to a difference in the research results by affecting individual compliance with a chatbot.

The findings of this systematic review showed that NLP chatbot–based intervention had a positive impact on the alteration of adults’ smoking behaviors. Regarding the effective role of chatbots in smoking cessation, a systematic review using meta-analysis indicated that at the 6-month follow-up, participants (aged 15 years and older) who received chatbot-based interventions were significantly more likely to quit smoking than those in the control group [15], supporting the findings derived from our review.

Our review revealed that participants had a very positive impression of chatbot use, feeling chatbots are useful, credible, and financially feasible. Similarly, a review indicated that participants emphasized numerous positive aspects of chatbots, especially their unique personalities and the capacity to offer empathetic and emotional support. However, several limitations were also pointed out. For instance, chatbots often had trouble fully understanding users, their responses were repetitive, and they lacked interactivity [18]. Moreover, another review showed that among the 5 studies included, only 2 were content with the application of chatbots in the intervention [37]. We assume that the user experience of participants is closely related to the performance of specific chatbots, such as their language comprehension, interaction, empathy, and persuasion abilities. NLP chatbots that are capable of free dialogue have an advantage.

We conducted a comprehensive literature search of preprinted, unpublished, and published records based on the preregistered study protocol. The inclusion criteria of this review were clear and strict (RCT only or NLP-chatbot only) which reached a high hierarchy of evidence. We analyzed 7 RCTs focusing on diet, physical activity, and tobacco smoking behaviors to explore the effectiveness of chatbot intervention. In this review, in addition to indicators of chatbot-related behavior change, we also paid attention to multiple secondary outcomes to explore an individual’s acceptability of chatbot intervention. No change was made to methods when compared to our study protocol.

However, the following limitations need to be noted when interpreting the findings. Few included studies measured user acceptance of chatbot intervention or the promoting or hindering factors of them to use chatbots. This impeded us from further studying the specific mechanism between the population’s use of chatbots and the change of related behaviors. There is little literature on privacy issues when using chatbots, which we thought was a serious and significant issue to consider. Besides, about half of the included studies had an elevated overall risk of bias.

Based on this review, we had some suggestions for future research. Chatbots are just a form and carrier of intervention, essentially requiring the support of various behavioral change theories. Future research would better incorporate appropriate theoretical frameworks, such as motivational interviewing theory [38] and the transtheoretical model [39] when designing chatbots to better achieve the goal of promoting health. Most of the included studies focused on a single chatbot intervention through voice conversation and message exchange in the intervention group, limiting the potential applications of chatbots. Future studies can integrate chatbots with other interventions to augment compliance with other interventions. Approximately half of the included studies did not describe the acceptability of NLP chatbot–based interventions. From the perspective of research implementation, future research needs to pay more attention to process evaluation, such as frequency and time of chatbot use. In terms of research outcome, future research needs to evaluate both outcome indicators and mediators, such as knowledge, motivation, and intention, to explore the deep reasons for the behavior change of participants after the chatbot intervention.

### Conclusion

Our results indicated that NLP-chatbots were promising in reducing tobacco smoking among adults, while their effects on the changes in dietary and physical activity behaviors remained inconclusive. Future research can be improved in aspects such as increasing the theoretical support for interventions and monitoring the interaction between users and NLP-chatbots.

## Supplementary material

10.2196/66403 Multimedia Appendix 1 Search terms for databases.

10.2196/66403 Multimedia Appendix 2 Table S1: characteristics and outcomes. 66403-1122796-1-SP.xlsx

10.2196/66403 Checklist 1 PRISMA checklist. PRISMA: Preferred Reporting Items for Systematic Reviews and Meta-Analyses.

## Abbreviations

AI: artificial intelligence
E: effective
NE: not effective
NLP: natural language processing
PE: probably effective
PNE: not to be probably effective
PRISMA: Preferred Reporting Items for Systematic Reviews and Meta-Analyses
PROSPERO: International Prospective Register of Systematic Reviews
RCT: randomized controlled trial
RoB: Risk of Bias
