# Supplementary material for: Natural Language Processing Chatbot–Based Interventions for Improvement of Diet, Physical Activity, and Tobacco Smoking Behaviors: Systematic Review
Source: JMIR Mhealth Uhealth. 2025 Jun 11;13:e66403. doi: 10.2196/66403 (PMC12175970; doi:10.2196/66403)
Supplement: Multimedia Appendix 1 [file mhealth-v13-e66403-s001.pdf]

1. PubMed

#	Searches
1	("Ai agent" OR "Ai agents" OR "Artificial agent" OR "Artificial agents" OR Artificial intelligence chatbot OR Artificial intelligence chatbots OR "Assistance technology" OR "Assistance technologies" OR "Chat bot" OR "Chat bots" OR Chatbot OR Chatbots OR Chatterbot OR "Conversational agent" OR "Conversational agents" OR "Conversational AI" OR "Conversational assistant" OR "Conversational assistants" OR Conversational bot OR Conversational bots OR "Conversational interface" OR "Conversational interfaces" OR "Conversational system" OR "Conversational systems" OR "Dialog system" OR "Dialog systems" OR Dialogue agent OR "Dialogue system" OR "Dialogue systems" OR "Digital assistant" OR "Digital assistants" OR Infobot OR "Intelligent agent" OR "Intelligent agents" OR Intelligent conversational assistant OR "Interactive agent" OR "Interactive agents" OR "Relational agent" OR "Relational agents" OR "Social robot" OR "Social robots" OR Virtual advisors OR "Virtual agent" OR "Virtual agents" OR "Virtual assistant" OR "Virtual assistants" OR "Virtual avatar" OR "Virtual avatars" OR "Virtual coach" OR "Virtual coaches" OR Virtual conversational agents)
2	(Diet [mh] OR Diet [tiab] OR Dietary[tiab] OR Exercise [mh] OR Exercise [tiab] OR Lifestyle [mh] OR Lifestyle [tiab] OR "life style" [tiab] OR "life styles"[tiab]OR Nutrition [mh] OR nutrition [tiab] OR "Physical activity" [tiab] OR "Physical activities"[tiab] OR "Sedentary behavior"[mh] OR "Sedentary behavior" [tiab] OR "Sedentary time" [tiab] OR sitting [tiab] OR "weight control" [tiab] OR "Weight loss" [mh] OR "Weight loss" [tiab] OR "Weight losses"[tiab] OR "Weight maintenance"[tiab] OR "smoking cessation"[mh] OR "smoking cessation"[tiab] OR "smoking cessations"[tiab] OR "smoking reduction"[mh] OR "smoking reduction"[tiab] OR "smoking habit"[tiab] OR "smoking habits"[tiab] OR "Smoking Prevention"[mh] OR "smoking prevention"[tiab] OR "tobacco control"[mh] OR "quit smoking"[tiab] OR "stop smoking"[tiab] OR "smoking prevention"[tiab] OR "health behavior"[mh] OR "health behavior"[tiab] OR "cigarette smoking")
3	#1 AND #2<br>Filters applied: from 2010/1/1 - 3000/12/12

2. EMBASE

#	Searches
1	'ai agent' OR 'ai agents' OR 'artificial agent' OR 'artificial agents' OR 'artificial intelligence chatbot' OR 'artificial intelligence chatbots' OR 'assistance technology' OR 'assistance technologies' OR 'chat bot' OR 'chat bots' OR chatbot OR chatbots OR chatterbot OR 'conversational agent' OR 'conversational agents' OR 'conversational ai' OR 'conversational assistant' OR 'conversational assistants' OR 'conversational bot' OR 'conversational bots' OR 'conversational interface' OR 'conversational interfaces' OR 'conversational system' OR 'conversational systems' OR 'dialog system' OR 'dialog systems' OR 'dialogue agent' OR 'dialogue system' OR 'dialogue systems' OR 'digital assistant' OR 'digital assistants' OR infobot OR 'intelligent agent' OR 'intelligent agents' OR 'intelligent conversational assistant' OR 'interactive agent' OR 'interactive agents' OR 'relational agent' OR 'relational agents' OR 'social robot' OR 'social robots' OR 'virtual advisors' OR 'virtual agent' OR 'virtual agents' OR 'virtual assistant' OR 'virtual assistants' OR 'virtual avatar' OR 'virtual avatars' OR 'virtual coach' OR 'virtual coaches' OR 'virtual conversational agents'
2	'body weight control'/exp OR 'body weight control' OR 'body weight loss'/exp OR 'body weight loss' OR 'diet'/exp OR 'diet' OR dietary OR 'nutrition'/exp OR 'nutrition' OR 'physical activity, capacity and performance'/exp OR 'physical activity'/exp OR 'physical activity' OR 'physical activities' OR 'exercise'/exp OR 'exercise' OR 'lifestyle'/exp OR 'lifestyle' OR 'lifestyle modification'/exp OR 'lifestyle modification' OR 'life style'/exp OR 'life style' OR lifestyle OR lifestyles OR 'sedentary time'/exp OR 'sedentary time' OR 'sedentary lifestyle'/exp OR 'sedentary lifestyle' OR 'sitting'/exp OR 'sitting' OR 'smoking cessation'/exp OR 'smoking cessation' OR 'smoking reduction'/exp OR 'smoking reduction' OR 'smoking and smoking related phenomena'/exp OR 'smoking'/exp OR Cigarette OR Tobacco OR Smoking OR (((quit$ or stop$ or ceas$ or giv$ or prevent$) adj3 smok$) or cigarette$):ti,ab
3	#1 AND #2<br>[2010-2023]/py

3. ACM Digital Library (The ACM Guide to Computing Literature)

#	**Search by title, keyword and abstract**
1	("ai agent" OR "ai agents" OR "artificial agent" OR "artificial agents" OR "artificial intelligence chatbot" OR "artificial intelligence chatbots" OR "assistance technology" OR "assistance technologies" OR "chat bot" OR "chat bots" OR chatbot OR chatbots OR chatterbot OR "conversational agent" OR "conversational agents" OR "conversational ai" OR "conversational assistant" OR "conversational assistants" OR "conversational bot" OR "conversational bots" OR "conversational interface" OR "conversational interfaces" OR "conversational system" OR "conversational systems" OR "dialog system" OR "dialog systems" OR "dialogue agent" OR "dialogue agents" OR "dialogue system" OR "dialogue systems" OR "digital assistant" OR "digital assistants" OR infobot OR "intelligent agent" OR "intelligent agents" OR "intelligent conversational assistant" OR "interactive agent" OR "interactive agents" OR "relational agent" OR "relational agents" OR "social robot" OR "social robots" OR "virtual advisors" OR "virtual agent" OR "virtual agents" OR "virtual assistant" OR "virtual assistants" OR "virtual avatar" OR "virtual avatars" OR "virtual coach" OR "virtual coaches" OR "virtual conversational agents")
2	(Diet OR dietary OR exercise OR exercises OR "healthy eating" OR lifestyle OR lifestyles OR "life style" OR "life styles" OR nutrition OR "physical activity" OR "physical activities" OR "sedentary behavior "OR "sedentary time" OR sitting OR "weight loss" OR "weight losses" OR "weight maintenance" OR "weight control" OR "smoking cessation" OR "smoking cessations" OR "smoking reduction" OR "tobacco" OR "cigarette" OR "smoking habit" OR "smoking habits" OR "smoking prevention" OR "health behavior")
3	#1 AND #2 search by title<br>#1 AND #2 search by keyword<br>#1 AND #2 search by abstract<br>Since 2010

4. Web of Science Core Collection

#	**Topic Searches**
1	("Ai agent" OR "Ai agents" OR "Artificial agent" OR "Artificial agents" OR "Artificial intelligence chatbot" OR "Artificial intelligence chatbots" OR "Assistance technology" OR "Assistance technologies" OR "Chat bot" OR "Chat bots" OR Chatbot OR Chatbots OR Chatterbot OR "Conversational agent" OR "Conversational agents" OR "Conversational AI" OR "Conversational assistant" OR "Conversational assistants" OR "Conversational bot" OR "Conversational bots" OR "Conversational interface" OR "Conversational interfaces" OR "Conversational system" OR "Conversational systems" OR "Dialog system" OR "Dialog systems" OR "Dialogue agent" OR "Dialogue agents" OR "Dialogue system" OR "Dialogue systems" OR "Digital assistant" OR "Digital assistants" OR Infobot OR "Intelligent agent" OR "Intelligent agents" OR "Intelligent conversational assistant" OR "Interactive agent" OR "Interactive agents" OR "Relational agent" OR "Relational agents" OR "Social robot" OR "Social robots" OR "Virtual advisors" OR "Virtual agent" OR "Virtual agents" OR "Virtual assistant" OR "Virtual assistants" OR "Virtual avatar" OR "Virtual avatars" OR "Virtual coach" OR "Virtual coaches" OR "Virtual conversational agents")
2	(Diet OR dietary OR exercise OR exercises OR "healthy eating" OR lifestyle OR lifestyles OR "life style" OR "life styles" OR nutrition OR "physical activity" OR "physical activities" OR "sedentary behavior" OR "sedentary time" OR sitting OR "weight loss" OR "weight losses" OR "weight maintenance" OR "weight control" OR "smoking cessation" OR "smoking cessations" OR "smoking reduction" OR "tobacco" OR "cigarette" OR "smoking habit" OR "smoking habits" OR "smoking prevention" OR "health behavior")
	#1 AND #2 topic searches<br>Timespan: 2010-01-01 to 2023-07-14 (Index Date)

5. PsycINFO

#	Searches
1	"Ai agent" OR "Ai agents" OR "Artificial agent" OR "Artificial agents" OR "Artificial intelligence chatbots" OR "Assistance technology" OR "Assistance technologies" OR "Chat bot" OR "Chat bots" OR IF (Chatbot) OR Chatbots OR Chatterbot OR MAINSUBJECT.EXACT("Conversational Agents") OR "Conversational AI" OR "Conversational assistant" OR "Conversational assistants" OR "Conversational bot" OR "Conversational bots" OR "Conversational interface" OR "Conversational interfaces" OR "Conversational system" OR "Conversational systems" OR "Dialog system" OR "Dialog systems" OR "Dialogue agent" OR "Dialogue system" OR "Dialogue systems" OR "Digital assistant" OR "Digital assistants" OR MAINSUBJECT.EXACT("Human Robot Interaction") OR Infobot OR MAINSUBJECT.EXACT("Intelligent Agents") OR "Intelligent conversational assistant" OR "Interactive agent" OR "Interactive agents" OR "Relational agent" OR "Relational agents" OR MAINSUBJECT.EXACT("Social Robotics") OR "Social robots" OR "Virtual advisors" OR "Virtual agent" OR "Virtual agents" OR "Virtual assistant" OR "Virtual assistants" OR "Virtual avatar" OR "Virtual avatars" OR "Virtual coach" OR "Virtual coaches" OR "Virtual conversational agents"
2	MAINSUBJECT.EXACT("Diets") OR diet OR dietary OR MAINSUBJECT.EXACT("Eating Behavior") OR MAINSUBJECT.EXACT("Exercise") OR MAINSUBJECT.EXACT("Health Behavior") OR MAINSUBJECT.EXACT("Lifestyle") OR lifestyles OR MAINSUBJECT.EXACT("Lifestyle Changes") OR MAINSUBJECT.EXACT("Nutrition") OR MAINSUBJECT.EXACT("Physical Activity") OR "physical activities" OR MAINSUBJECT.EXACT("Physical Fitness") OR MAINSUBJECT.EXACT("Sedentary Behavior") OR sitting OR MAINSUBJECT.EXACT.EXPLODE("Weight Control") OR MAINSUBJECT.EXACT("Weight Loss" OR "weight maintenance") OR MAINSUBJECT.EXACT("Smoking cessation") OR "smoking cessations" OR MAINSUBJECT.EXACT("Smoking prevention") OR "smoking prevention" OR MAINSUBJECT.EXACT("Tobacco control") OR MAINSUBJECT.EXACT("Passive smoking ") OR MAINSUBJECT.EXACT("Nicotine withdrawal") OR smoking OR "smoking habit" OR tobacco OR nicotine
3	#1 AND #2<br>Publication Date: 20100101-

6. IEEE

#	Searches
1	"Ai agent" OR "Ai agents" OR "Artificial agent" OR "Artificial agents" OR "Artificial intelligence chatbot" OR "Artificial intelligence chatbots" OR "Assistance technology" OR "Assistance technologies" OR "Chat bot" OR "Chat bots" OR Chatbot OR Chatbots OR Chatterbot OR "Conversational agent" OR "Conversational agents" OR "Conversational AI" OR "Conversational assistant" OR "Conversational assistants" OR "Conversational bot" OR "Conversational bots" OR "Conversational interface" OR "Conversational interfaces" OR "Conversational system" OR "Conversational systems" OR "Dialog system" OR "Dialog systems" OR "Dialogue agent" OR "Dialogue system" OR "Dialogue systems" OR "Digital assistant" OR "Digital assistants" OR Infobot OR "Intelligent agent" OR "Intelligent agents" OR "Intelligent conversational assistant" OR "Interactive agent" OR "Interactive agents" OR "Relational agent" OR "Relational agents" OR "Social robot" OR "Social robots" OR "Virtual advisors" OR "Virtual agent" OR "Virtual agents" OR "Virtual assistant" OR "Virtual assistants" OR "Virtual avatar" OR "Virtual avatars" OR "Virtual coach" OR "Virtual coaches" OR "Virtual conversational agents"
2	Diet OR dietary OR exercise OR exercises OR "healthy eating" OR lifestyle OR lifestyles OR "life style" OR "life styles" OR nutrition OR "physical activity" OR "physical activities" OR "sedentary behavior" OR "sedentary time" OR sitting OR "weight loss" OR "weight losses" OR "weight maintenance" OR "weight control" OR "smoking cessation" OR "smoking cessations" OR "smoking reduction" OR "tobacco" OR "cigarette" OR "smoking habit" OR "smoking habits" OR "smoking prevention" OR "health behavior"
3	#1 AND #2<br>Specify Year Range: 2010-2024<br>All Metadata

("All Metadata":"Ai agent" OR "All Metadata":"Ai agents" OR "All Metadata":"Artificial agent" OR "All Metadata":"Artificial agents" OR "All Metadata":"Artificial intelligence chatbot" OR "All Metadata":"Artificial intelligence chatbots" OR "All Metadata":"Assistance technology" OR "All Metadata":"Assistance technologies" OR "All Metadata":"Chat bot" OR "All Metadata":"Chat bots" OR "All Metadata":Chatbot OR "All Metadata":Chatterbot OR "All Metadata":"Conversational agent" OR "All Metadata":"Conversational agents" OR "All Metadata":"Conversational AI" OR "All Metadata":"Conversational assistant" OR "All Metadata":"Conversational assistants" OR "All Metadata":"Conversational bot" OR "All Metadata":"Conversational bots" OR "All

Metadata":"Conversational interface" OR "All Metadata":"Conversational interfaces" OR "All Metadata":"Conversational system" OR "All Metadata":"Conversational systems" OR "All Metadata":"Dialog system" OR "All Metadata":"Dialog systems" OR "All Metadata":"Dialogue agent" OR "All Metadata":"Dialogue system" OR "All Metadata":"Dialogue systems" OR "All Metadata":"Digital assistant" OR "All Metadata":"Digital assistants" OR "All Metadata":Infobot OR "All Metadata":"Intelligent agent" OR "All Metadata":"Intelligent agents" OR "All Metadata":"Intelligent conversational assistant" OR "All Metadata":"Interactive agent" OR "All Metadata":"Interactive agents" OR "All Metadata":"Relational agent" OR "All Metadata":"Relational agents" OR "All Metadata":"Social robot" OR "All Metadata":"Social robots" OR "All Metadata":"Virtual advisors" OR "All Metadata":"Virtual agent" OR "All Metadata":"Virtual agents" OR "All Metadata":"Virtual assistant" OR "All Metadata":"Virtual assistants" OR "All Metadata":"Virtual avatar" OR "All Metadata":"Virtual avatars" OR "All Metadata":"Virtual coach" OR "All Metadata":"Virtual coaches" OR "All Metadata":"Virtual conversational agents") AND ("All Metadata":Diet OR "All Metadata":dietary OR "All Metadata":exercise OR "All Metadata":exercises OR "All Metadata":"healthy eating" OR "All Metadata":lifestyle OR "All Metadata":lifestyles OR "All Metadata":"life style" OR "All Metadata":"life styles" OR "All Metadata": nutrition OR "All Metadata":"physical activity" OR "All Metadata":"physical activities" OR "All Metadata":"sedentary behavior" OR "All Metadata":"sedentary time" OR "All Metadata":sitting OR "All Metadata":"weight loss" OR "All Metadata":"weight losses" OR "All Metadata":"weight maintenance" OR "All Metadata":"weight control" OR "All Metadata":"smoking cessation" OR "All Metadata":"smoking cessations" OR "All Metadata":"smoking reduction" OR "All Metadata":"tobacco" OR "All Metadata":"cigarette" OR "All Metadata":"smoking habit" OR "All Metadata":"smoking habits" OR "All Metadata":"smoking prevention" OR "All Metadata":"health behavior")

7. CINAHL Plus with Full Text

#	Searches
1	"Ai agent" OR "Ai agents" OR "Artificial agent" OR "Artificial agents" OR "Artificial intelligence chatbots" OR "Assistance technology" OR "Assistance technologies" OR "Chat bot" OR "Chat bots" OR IF (Chatbot) OR Chatbots OR Chatterbot OR MAINSUBJECT.EXACT("Conversational Agents") OR "Conversational AI" OR "Conversational assistant" OR "Conversational assistants" OR "Conversational bot" OR "Conversational bots" OR "Conversational interface" OR "Conversational interfaces" OR "Conversational system" OR "Conversational systems" OR "Dialog system" OR "Dialog systems" OR "Dialogue agent" OR "Dialogue system" OR "Dialogue systems" OR "Digital assistant" OR "Digital assistants" OR MAINSUBJECT.EXACT("Human Robot Interaction") OR Infobot OR MAINSUBJECT.EXACT("Intelligent Agents") OR "Intelligent conversational assistant" OR "Interactive agent" OR "Interactive agents" OR "Relational agent" OR "Relational agents" OR MAINSUBJECT.EXACT("Social Robotics") OR "Social robots" OR "Virtual advisors" OR "Virtual agent" OR "Virtual agents" OR "Virtual assistant" OR "Virtual assistants" OR "Virtual avatar" OR "Virtual avatars" OR "Virtual coach" OR "Virtual coaches" OR "Virtual conversational agents"
2	MAINSUBJECT.EXACT("Diets") OR diet OR dietary OR MAINSUBJECT.EXACT("Eating Behavior") OR MAINSUBJECT.EXACT("Exercise") OR MAINSUBJECT.EXACT("Health Behavior") OR MAINSUBJECT.EXACT("Lifestyle") OR lifestyles OR MAINSUBJECT.EXACT("Lifestyle Changes") OR MAINSUBJECT.EXACT("Nutrition") OR MAINSUBJECT.EXACT("Physical Activity") OR "physical activities" OR MAINSUBJECT.EXACT("Physical Fitness") OR MAINSUBJECT.EXACT("Sedentary Behavior") OR sitting OR MAINSUBJECT.EXACT.EXPLODE("Weight Control") OR MAINSUBJECT.EXACT("Weight Loss" OR "weight maintenance") OR MAINSUBJECT.EXACT("Smoking cessation") OR "smoking cessations" OR MAINSUBJECT.EXACT("Smoking prevention") OR "smoking prevention" OR MAINSUBJECT.EXACT("Tobacco control") OR MAINSUBJECT.EXACT("Passive smoking ") OR MAINSUBJECT.EXACT("Nicotine withdrawal") OR smoking OR "smoking habit" OR tobacco OR nicotine
3	#1 AND #2<br>Publication Date: 20100101-

8. Cochrane Library

#	**Search by title, keyword and abstract**
1	("Ai agent" OR "Ai agents" OR "Artificial agent" OR "Artificial agents" OR "Artificial intelligence chatbot" OR "Artificial intelligence chatbots" OR "Assistance technology" OR "Assistance technologies" OR "Chat bot" OR "Chat bots" OR Chatbot OR Chatbots OR Chatterbot OR "Conversational agent" OR "Conversational agents" OR "Conversational AI" OR "Conversational assistant" OR "Conversational assistants" OR "Conversational bot" OR "Conversational bots" OR "Conversational interface" OR "Conversational interfaces" OR "Conversational system" OR "Conversational systems" OR "Dialog system" OR "Dialog systems" OR "Dialogue agent" OR "Dialogue system" OR "Dialogue systems" OR "Digital assistant" OR "Digital assistants" OR Infobot OR "Intelligent agent" OR "Intelligent agents" OR "Intelligent conversational assistant" OR "Interactive agent" OR "Interactive agents" OR "Relational agent" OR "Relational agents" OR "Social robot" OR "Social robots" OR "Virtual advisors" OR "Virtual agent" OR "Virtual agents" OR "Virtual assistant" OR "Virtual assistants" OR "Virtual avatar" OR "Virtual avatars" OR "Virtual coach" OR "Virtual coaches" OR "Virtual conversational agents")
2	(Diet OR dietary OR exercise OR exercises OR "healthy eating" OR lifestyle OR lifestyles OR "life style" OR "life styles" OR nutrition OR "physical activity" OR "physical activities" OR "sedentary behavior" OR "sedentary time" OR sitting OR "weight loss" OR "weight losses" OR "weight maintenance" OR "weight control" OR "smoking cessation" OR "smoking cessations" OR "smoking reduction" OR "tobacco" OR "cigarette" OR "smoking habit" OR "smoking habits" OR "smoking prevention" OR "health behavior")
3	#1 AND #2 Title, Abstract, Keyword searches<br>Custom Range: 01/01/2010 to -

9. Scopus

#	**Search by title, keyword and abstract**
1	("Ai agent" OR "Ai agents" OR "Artificial agent" OR "Artificial agents" OR "Artificial intelligence chatbot" OR "Artificial intelligence chatbots" OR "Assistance technology" OR "Assistance technologies" OR "Chat bot" OR "Chat bots" OR Chatbot OR Chatbots OR Chatterbot OR "Conversational agent" OR "Conversational agents" OR "Conversational AI" OR "Conversational assistant" OR "Conversational assistants" OR "Conversational bot" OR "Conversational bots" OR "Conversational interface" OR "Conversational interfaces" OR "Conversational system" OR "Conversational systems" OR "Dialog system" OR "Dialog systems" OR "Dialogue agent" OR "Dialogue system" OR "Dialogue systems" OR "Digital assistant" OR "Digital assistants" OR Infobot OR "Intelligent agent" OR "Intelligent agents" OR "Intelligent conversational assistant" OR "Interactive agent" OR "Interactive agents" OR "Relational agent" OR "Relational agents" OR "Social robot" OR "Social robots" OR "Virtual advisors" OR "Virtual agent" OR "Virtual agents" OR "Virtual assistant" OR "Virtual assistants" OR "Virtual avatar" OR "Virtual avatars" OR "Virtual coach" OR "Virtual coaches" OR "Virtual conversational agents")
2	(Diet OR dietary OR exercise OR exercises OR "healthy eating" OR lifestyle OR lifestyles OR "life style" OR "life styles" OR nutrition OR "physical activity" OR "physical activities" OR "sedentary behavior" OR "sedentary time" OR sitting OR "weight loss" OR "weight losses" OR "weight maintenance" OR "weight control" OR "smoking cessation" OR "smoking cessations" OR "smoking reduction" OR "tobacco" OR "cigarette" OR "smoking habit" OR "smoking habits" OR "smoking prevention" OR "health behavior")
3	#1 AND #2 Title, Abstract, Keyword searches<br>2010-2023

10. Library, Information Science & Technology Abstracts

#	Searches
1	"Ai agent" OR "Ai agents" OR "Artificial agent" OR "Artificial agents" OR "Artificial intelligence chatbots" OR "Assistance technology" OR "Assistance technologies" OR "Chat bot" OR "Chat bots" OR IF (Chatbot) OR Chatbots OR Chatterbot OR MAINSUBJECT.EXACT("Conversational Agents") OR "Conversational AI" OR "Conversational assistant" OR "Conversational assistants" OR "Conversational bot" OR "Conversational bots" OR "Conversational interface" OR "Conversational interfaces" OR "Conversational system" OR "Conversational systems" OR "Dialog system" OR "Dialog systems" OR "Dialogue agent" OR "Dialogue system" OR "Dialogue systems" OR "Digital assistant" OR "Digital assistants" OR MAINSUBJECT.EXACT("Human Robot Interaction") OR Infobot OR MAINSUBJECT.EXACT("Intelligent Agents") OR "Intelligent conversational assistant" OR "Interactive agent" OR "Interactive agents" OR "Relational agent" OR "Relational agents" OR MAINSUBJECT.EXACT("Social Robotics") OR "Social robots" OR "Virtual advisors" OR "Virtual agent" OR "Virtual agents" OR "Virtual assistant" OR "Virtual assistants" OR "Virtual avatar" OR "Virtual avatars" OR "Virtual coach" OR "Virtual coaches" OR "Virtual conversational agents"
2	MAINSUBJECT.EXACT("Diets") OR diet OR dietary OR MAINSUBJECT.EXACT("Eating Behavior") OR MAINSUBJECT.EXACT("Exercise") OR MAINSUBJECT.EXACT("Health Behavior") OR MAINSUBJECT.EXACT("Lifestyle") OR lifestyles OR MAINSUBJECT.EXACT("Lifestyle Changes") OR MAINSUBJECT.EXACT("Nutrition") OR MAINSUBJECT.EXACT("Physical Activity") OR "physical activities" OR MAINSUBJECT.EXACT("Physical Fitness") OR MAINSUBJECT.EXACT("Sedentary Behavior") OR sitting OR MAINSUBJECT.EXACT.EXPLODE("Weight Control") OR MAINSUBJECT.EXACT("Weight Loss" OR "weight maintenance") OR MAINSUBJECT.EXACT("Smoking cessation") OR "smoking cessations" OR MAINSUBJECT.EXACT("Smoking prevention") OR "smoking prevention" OR MAINSUBJECT.EXACT("Tobacco control") OR MAINSUBJECT.EXACT("Passive smoking ") OR MAINSUBJECT.EXACT("Nicotine withdrawal") OR smoking OR "smoking habit" OR tobacco OR nicotine
3	#1 AND #2<br>Publication Date: 20100101-20231231

11. ClinicalTrials.gov

#	Searches
1<br>Intervention/Treatment	("Ai agent" OR "Ai agents" OR "Artificial agent" OR "Artificial agents" OR "Artificial intelligence chatbot" OR "Artificial intelligence chatbots" OR "Assistance technology" OR "Assistance technologies" OR "Chat bot" OR "Chat bots" OR Chatbot OR Chatbots OR Chatterbot OR "Conversational agent" OR "Conversational agents" OR "Conversational AI" OR "Conversational assistant" OR "Conversational assistants" OR "Conversational bot" OR "Conversational bots" OR "Conversational interface" OR "Conversational interfaces" OR "Conversational system" OR "Conversational systems" OR "Dialog system" OR "Dialog systems" OR "Dialogue agent" OR "Dialogue system" OR "Dialogue systems" OR "Digital assistant" OR "Digital assistants" OR Infobot OR "Intelligent agent" OR "Intelligent agents" OR "Intelligent conversational assistant" OR "Interactive agent" OR "Interactive agents" OR "Relational agent" OR "Relational agents" OR "Social robot" OR "Social robots" OR "Virtual advisors" OR "Virtual agent" OR "Virtual agents" OR "Virtual assistant" OR "Virtual assistants" OR "Virtual avatar" OR "Virtual avatars" OR "Virtual coach" OR "Virtual coaches" OR "Virtual conversational agents")
2<br>Condition or disease	(Diet OR dietary OR exercise OR exercises OR "healthy eating" OR lifestyle OR lifestyles OR "life style" OR "life styles" OR nutrition OR "physical activity" OR "physical activities" OR "sedentary behavior" OR "sedentary time" OR sitting OR "weight loss" OR "weight losses" OR "weight maintenance" OR "weight control" OR "smoking cessation" OR "smoking cessations" OR "smoking reduction" OR "tobacco" OR "cigarette" OR "smoking habit" OR "smoking habits" OR "smoking prevention" OR "health behavior")
3	#1 AND #2<br>Study start From 01/01/2010

12. China National Knowledge Infrastructure (searched in Chinese)

#	Searches
1	聊天机器人 + 会话代理人
2	运动 + 体力活动 + 饮食 + 吸烟 + 减重 + 体重控制 + 生活方式
3	篇关摘、模糊搜索、中英文扩展<br>#1 AND #2<br>发表时间：2010-01-01
